# Precise base editing with CC context-specificity using engineered human APOBEC3G-nCas9 fusions

**DOI:** 10.1186/s12915-020-00849-6

**Published:** 2020-08-31

**Authors:** Zhiquan Liu, Siyu Chen, Huanhuan Shan, Yingqi Jia, Mao Chen, Yuning Song, Liangxue Lai, Zhanjun Li

**Affiliations:** 1 Key Laboratory of Zoonosis Research, Ministry of Education, College of Animal Science, Jilin University, Changchun, 130062 China; 2 CAS Key Laboratory of Regenerative Biology, Guangdong Provincial Key Laboratory of Stem Cell and Regenerative Medicine, South China Institute for Stem Cell Biology and Regenerative Medicine, Guangzhou Institutes of Biomedicine and Health, Chinese Academy of Sciences, Guangzhou, 510530 China; 3 Guangzhou Regenerative Medicine and Health Guang Dong Laboratory (GRMH-GDL), Guangzhou, 510005 China; 4 Institute for Stem Cell and Regeneration, Chinese Academy of Sciences, Beijing, 100101 China

**Keywords:** CRISPR/Cas9, Base editor, eA3G, Precision

## Abstract

**Background:**

Cytidine base editors (CBEs), composed of a cytidine deaminase fused to Cas9 nickase (nCas9), enable efficient C-to-T conversion in various organisms. However, current base editors can induce unwanted bystander C-to-T conversions when multiple Cs are present in the ~ 5-nucleotide activity window of cytidine deaminase, which negatively affects their precision. Here, we develop a new base editor which significantly reduces unwanted bystander activities.

**Results:**

We used an engineered human APOBEC3G (eA3G) C-terminal catalytic domain with preferential cytidine-deaminase activity in motifs with a hierarchy CCC>CCC>CC (where the preferentially deaminated C is underlined), to develop an eA3G-BE with distinctive CC context-specificity and reduced generation of bystander mutations. Targeted editing efficiencies of 18.3–58.0% and 54.5–92.2% with excellent CC context-specificity were generated in human cells and rabbit embryos, respectively. In addition, a base editor that can further recognize relaxed NG PAMs is achieved by combining hA3G with an engineered SpCas9-NG variant. The A3G-BEs were used to induce accurate single-base substitutions which led to nonsense mutation with an efficiency of 83–100% and few bystander mutations in Founder (F0) rabbits at *Tyr* loci.

**Conclusions:**

These novel base editors with improved precision and CC context-specificity will expand the toolset for precise gene modification in organisms.

## Background

The clustered regularly interspaced short palindromic repeat (CRISPR) system has exhibited powerful genome manipulation capability in various organisms [1]. Base editor, a revolutionary technology derived from the CRISPR system, is composed of a cytidine deaminase or an evolved adenine deaminase fused to nCas9 and enables the conversion of C·G to T·A or A·T to G·C base pair in organisms, respectively [2, 3]. In contrast to conventional gene-editing nucleases, CBE represents significant advances in precise genome manipulation since it can achieve targeted C-to-T conversions without generating DNA double-strand breaks (DSBs) or requiring a donor template, and it induces lower levels of unwanted insertion/deletion mutations (indels) [2, 4]. The most commonly used CBE architecture, rA1-BE, consists of rat APOBEC1 (rA1) fused to a *Streptococcus pyogenes* Cas9 (SpCas9) nickase [2]. Efficient editing by rA1-BE requires the target C within a ~ 5-nucleotide window near the protospacer-adjacent motif (PAM)-distal end of the protospacer (positions 4–8, counting the PAM as positions 21–23) in human cells [2]. The unwanted bystander C-to-T conversions will be generated when multiple Cs are present in the enzyme’s activity window [2, 5]. It negatively affects the precision of targeted base editing, which are not ideal for precise disease modeling and gene therapy where accurate single C substitution is required [6].

To overcome this limitation, optimized rA1 with mutant deaminase domains (YE base editors) or shortened linker between rA1 and nCas9 has been used to narrow the editing window in human cells [7, 8]. The representative YE systems termed YE1 (W90Y+R126E) and YEE (W90Y+R126E+R132E), to effectively narrow the width of the editing window from ~ 5 nucleotides to as little as ~ 1–2 nucleotides in human cells [7]. Additionally, YEE-BE showed better accuracy than YE1-BE, but has lowered editing efficiency at target loci [7, 9]. Moreover, an engineered human APOBEC3A (eA3A) domain with TCR (R = A/G) context-specificity has been reported to efficiently reduce bystander mutations, and it has been proven to be superior to conventional base editors with narrowed window in the TCR motifs [9, 10]. Context-dependent base editors, such as eA3A-BE, represent an important direction that offers precise base editing, while the application of them was restricted by the presence of TCR motifs [9]. Additionally, although many precise base editors exist currently, it is still difficult to achieve accurate editing in target sites with multiple Cs.

Previous study has shown the hA3G preferentially deaminates cytidines in CC and CCC motif in vitro [11–13]; thus, we speculated it would have the potential to be developed as a CC context-dependent base editor. In addition, hA3G-BE3 has been reported to induce C-to-T conversions in mammalian cells with lower efficiency compared with rA1-BE3 [14, 15], but no one has thoroughly evaluated its editing efficiency in CC context, for which it shows distinctive preference. Here, a new base editor, eA3G-BE, was developed for drastically reducing bystander mutations using an engineered hA3G C-terminal catalytic domain which preferentially deaminates cytidines in specific motifs according to a CCC>CCC>CC hierarchy firstly (where the preferentially deaminated C is underlined). Moreover, the further engineered variant, eA3G-NG, could be used to expand genome-targeting scope with NG PAMs. These new base editing tools provide a simple and efficient method for inducing precise single-nucleotide substitution with CC context-specificity.

## Results

### Characterization of eA3G-BE that selectively edits Cs with CC context-specificity

**Fig. 1:**
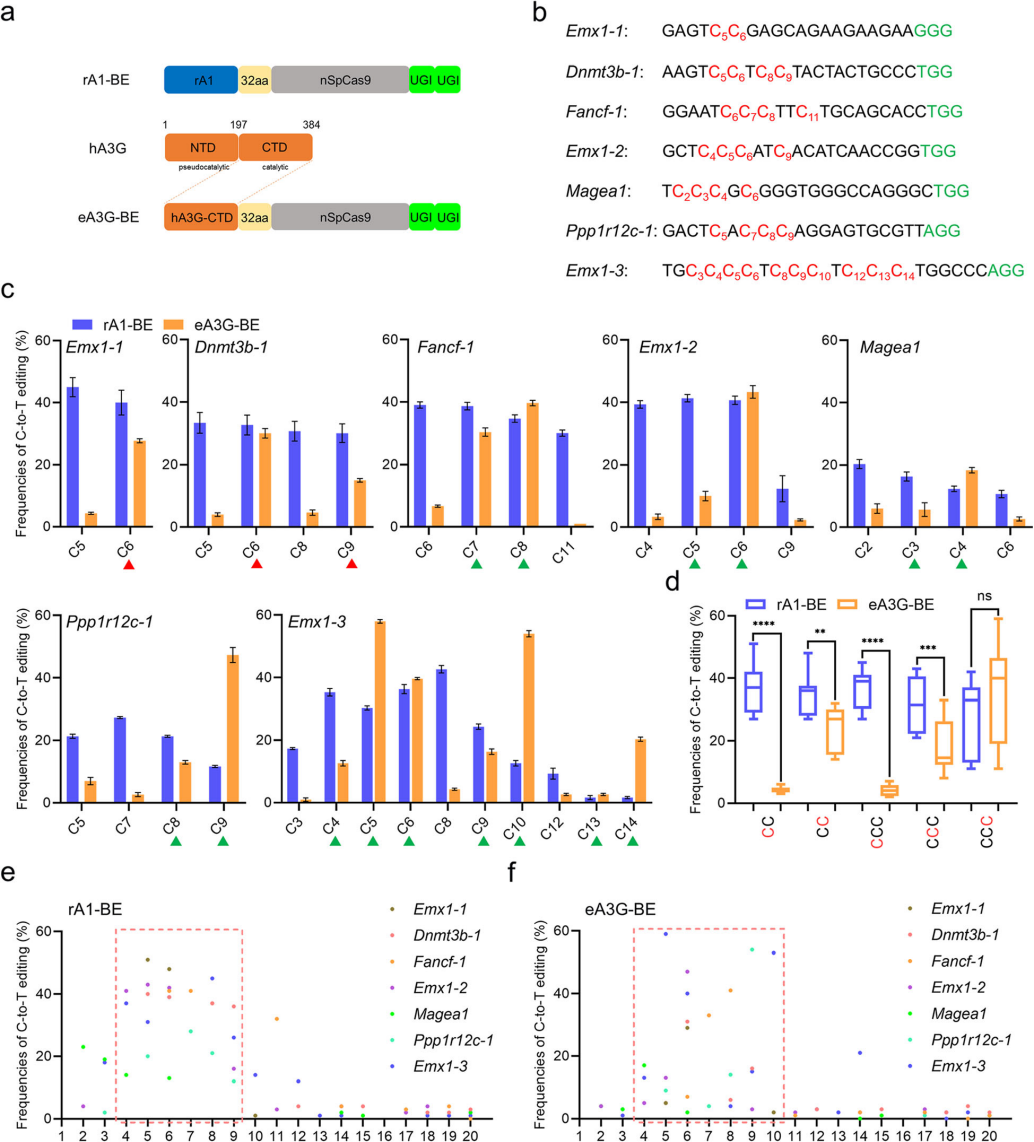
Base editing with CC context-specificity mediated by eA3G-BE in human cells. **a** Schematic representation of rA1-BE and eA3G-BE architecture. **b** Protospacers and PAM (green) sequences of the genomic loci tested, with the target Cs shown in red. Cytosines are counted with the base distal to the PAM setting as position 1. **c** Summary of C-to-T editing frequencies induced by rA1-BE and eA3G-BE systems on each cytosine at seven target sites. The eA3G-BE showed obvious preference at the cytosines in a CC (red triangle) or CCC (green triangle) context. **d** Effect of sequence context on base editing by rA1-BE and eA3G-BE (window 4–9). The frequencies were calculated using the data in Fig. 2c. **e**, **f** Summary of the base editing frequency at each cytosine in the spacer region for the indicated 7 sgRNAs using rA1-BE (**e**) and eA3G-BE (**f**). These data show that the major editing window ranges of rA1-BE and eA3G-BE from positions 4 to 9 or from positions 4 to10 in spacer region, respectively. Values and error bars reflect the mean ± s.e.m. of three independent biological replicates

The hA3G has a C-terminal catalytic domain (CTD) and a second pseudocatalytic domain at N-terminal which retains the same tertiary fold, but is not catalytically active [11]. To make use of its beneficial properties for base editing, we replaced rA1 with the engineered C-terminal catalytic domain of hA3G (hA3G-CTD) in rA1-BE4max [16], the current optimal architecture of CBE, to create eA3G-BE4max (referred to as eA3G-BE) (Fig. 1a).

To evaluate its general efficacy, we firstly tested both rA1-BE and eA3G-BE at 7 target sites with multiple Cs in human cells by co-transfecting them with the respective single guide RNAs (sgRNAs) into HEK293T cells (Fig. 1b). Western blotting demonstrated the protein products of eA3G-BE was comparable to that of classical rA1-BE (Additional file 1: Fig. S1). Base editing frequencies were evaluated from Sanger sequence chromatograms using EditR, a robust and inexpensive base editing quantification software [17]. Our results suggested that, compared with rA1-BE system, eA3G-BE showed a distinct preference for CC context in all seven tested sites (Fig. 1c). In two-C context, the eA3G-BE system has similar or lower editing efficiency at the second C (CC) but significantly reduced C-to-T conversions at the first C (CC) (Fig. 1c, d). Moreover, in more than three-C context, the eA3G-BE exhibited similar efficiency comparable to that of rA1-BE at the last C (CCC) (mean 37.0% vs. 28.8%, *P* = 0.055), meanwhile with lower efficiency at the middle C (CCC) (mean 17.4% vs. 31.4%, *P* = 0.0007) and significantly reduced efficiency at the first C (CCC) (mean 4.3% vs. 37.1%, *P* < 0.0001) (Fig. 1c, d). In addition, the significantly reduced base editing efficiencies of eA3G-BE were observed in 6 target sites with non-CC contexts compared with that of rA1-BE (Additional file 1: Fig. S2). The eA3G-BE can efficiently induce base editing in a major window (~ 7 nt, positions 4–10 in the sgRNA target site) compared with rA1-BE (~ 6 nt, positions 4–9), and it can even edit distal C14 at *Emx1-3* (Fig. 1e, f). Moreover, the eA3G-BE showed reduced off-target base editing compared with rA1-BE, consistent with previous report of eA3A-BE [9] (Additional file 1: Fig. S3). Taken together, these results suggested that the eA3G-BE can induce site-dependent lower or similar base editing efficiency compared to that of rA1-BE, meanwhile maintaining distinct preference for CC context in human cells.

### Comparison of base editing activities and precision among eA3G-BE and other precise BEs

**Fig. 2:**
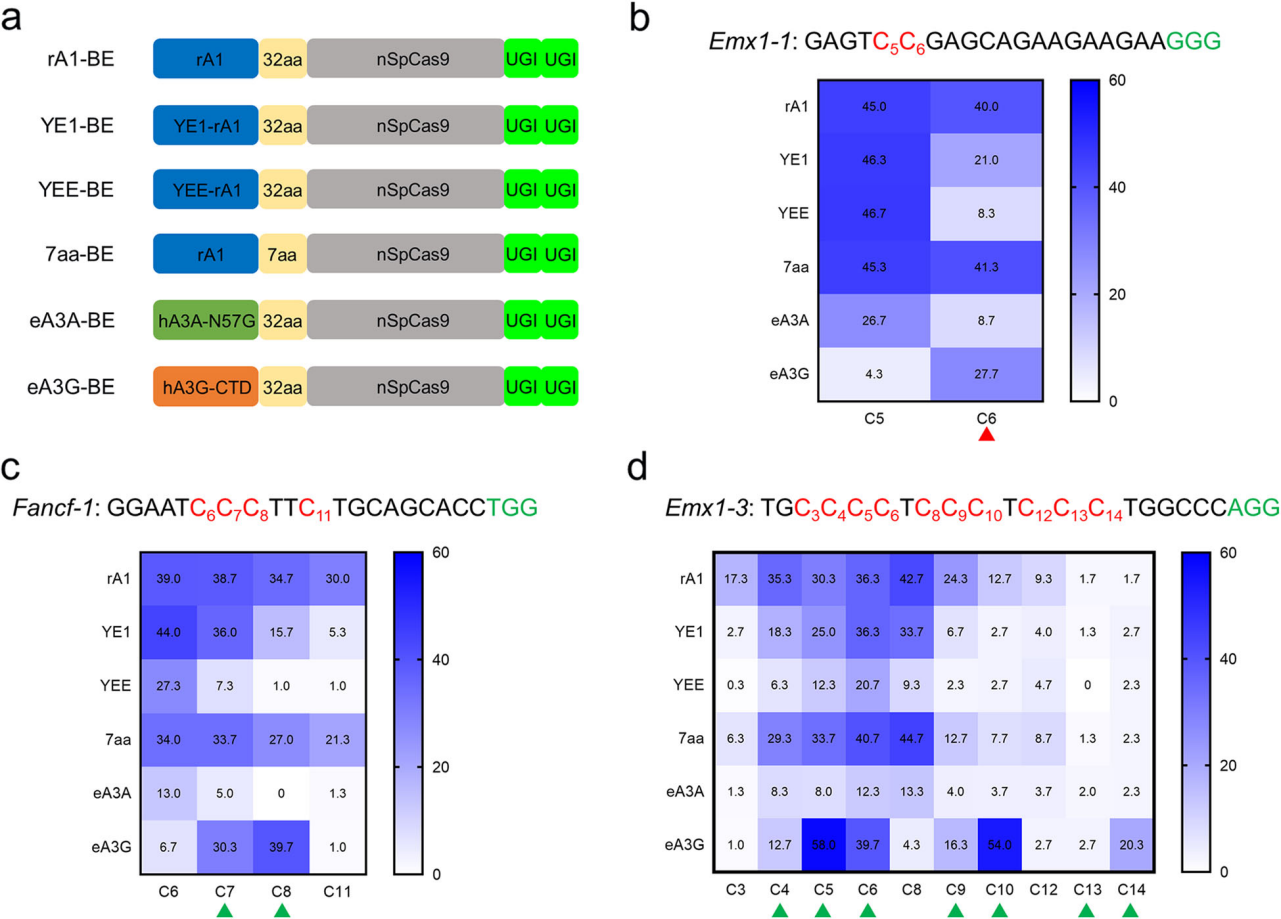
Comparison of base editing activity and precision using eA3G-BE and other precise BEs. **a** Schematic representation of six base editors’ architecture. YE1 (bearing W90Y+R126E mutations in rA1), YEE (bearing W90Y+R126E+R132E mutations in rA1), and eA3A (bearing N57G mutation in hA3A). **b**–**d** Heat maps showing C-to-T editing efficiencies for six base editors at *Emx1-1* (**b**), *Fancf* (**c**), and *Emx1-3*(**d**). The CC or CCC context was shown in red triangle or green triangle, respectively. Values and error bars reflect the mean ± s.e.m. of three independent biological replicates

To compare the precision of eA3G-BE with current representative precise BEs, we compared the editing activities of six base editor fusions at three representative target sites with multiple Cs: the original rA1-BE, the eA3G-BE, three rA1-BE variants, YE1 and YEE (YE BEs) which have mutations in rA1 designed to slow its kinetic rate so as to restrict the editing window, 7aa-BE with narrowed window by shortening the linker between the rA1 and the Cas9 domain, and eA3A-BE which bears N57G mutation in hA3A (Fig. 2a). Among the six base editors tested, eA3G-BE displayed the unique CC context-specificity, meanwhile minimizing bystander cytidine editing in non-CC context at all tested sites (Figs. 2b–d and Additional file 1: Fig. S4). Moreover, eA3G-BE showed lower activity at *Emx1-1* (mean 27.7%) with CC context, but highest activity at the third C (CCC) at *Fancf* (mean 39.7%) and *Emx1-3* (mean 58.0%) (Fig. 2b–d). YE BEs effectively narrow the editing window to ~ 1–2 nucleotides (mainly from C5 to C6) with similar or lower editing efficiency (Fig. 2b–d), consistent with previous study [7]. The eA3A-BE exhibited obvious preference for TC context, while it reduced base editing activity (Fig. 2b–d); it may be due to the fact that these three target sites were not classical TCR motifs [9]. However, no significantly narrowed editing window were observed using 7aa-BE (Fig. 2b–d), which is also consistent with previous reports that shortened linker did not substantially alter editing window [7, 9]. These results indicate that eA3G-BE system is efficient and precise in human cells with distinct CC context-specificity. In conjunction with previous precise BEs, eA3G-BE system could further enrich the tool kits for accurate gene editing and therapy.

### eA3G-BE can induce accurate C-to-T conversions in rabbit embryos

**Fig. 3:**
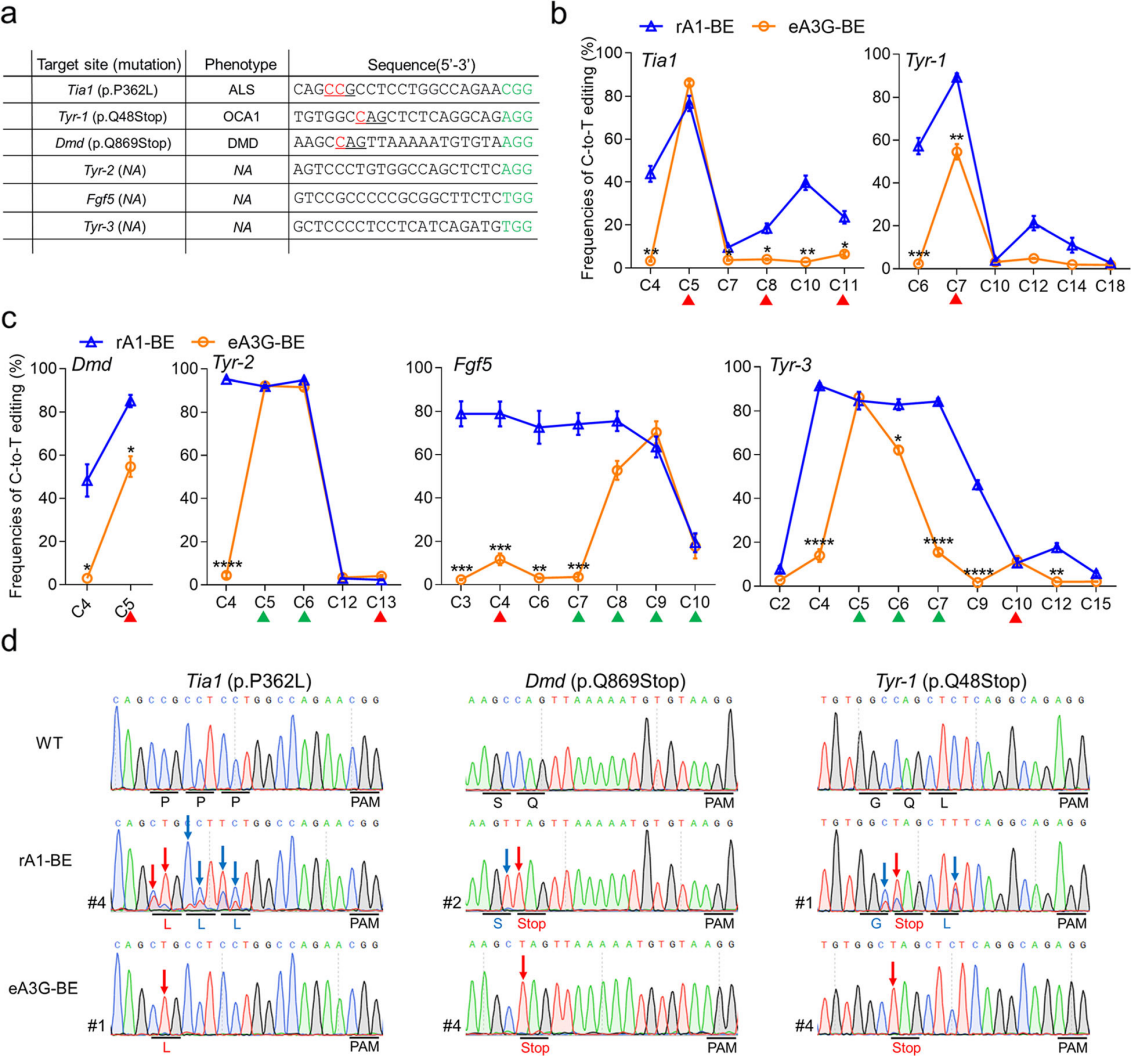
eA3G-BE can induce efficient C-to-T conversions with minimized bystander activity in rabbit embryos. **a** The six target-site sequences with CC context. Target sequence (black), PAM region (green), target base (red), and mutant amino acid (underlined). *NA*, not applicable. **b**, **c** Frequencies of single C-to-T conversions using rA1-BE and eA3G-BE at six sites in rabbit embryos. CC context (red triangle) and CCC context (green triangle). Target Cs are counting the PAM as positions 21–23. *n* = ~ 6 blastocysts. **d** Representative sequencing chromatograms of edited rabbit blastocyst at three target sites using rA1-BE and eA3G-BE systems. Targeted mutations (red arrows) and bystander mutations (blue arrows). The relevant codon identities at the target site are presented under the DNA sequence

To further evaluate the precision of eA3G-BE, six target sites including CC and CCC contexts were selected in rabbit embryos (Fig. 3a). Base editing was conducted in rabbit zygotes using microinjection of eA3G-encoding mRNA and single guide RNA (sgRNA). The control group, rA1-BE, showed a large editing window mainly from C3 to C9 (~ 7 nt) and even induced the widest range of mutations spanning from C2 to C15 at *Tyr-3* without obvious context-specificity (Fig. 3b, c). In contrast, eA3G-BE exhibited ideal efficiency comparable to that of rA1-BE at all six sites (average editing frequencies 54.5–92.2% vs. 76.6–95.2%) and significantly reduced bystander activities in non-CC contexts with a similar ~ 7 nt editing window (mainly from C4 to C10) (Fig. 3b, c). Moreover, the eA3G-BE exclusively edited the second C when the CC dinucleotide presents in the editing window, while with reduced efficiency at two (54.7 ± 10.7% vs. 85.1 ± 6.3%, *P* < 0.05 at *Dmd*; 54.5 ± 8.8% vs. 89.3 ± 4.2%, *P* < 0.01) of three tested sites compared with rA1-BE (Fig. 3b, c), consistent with that in human cells. In addition, at three tested sites with multiple Cs, eA3G-BE induced high editing frequencies in CCC (*Tyr-2* and *Tyr-3*) or CCC (*Fgf5*) contexts, meanwhile significantly decreased bystander activities at the first cytidine (Fig. 3c). Notably, with the high precision of eA3G-BE, targeted C-to-T conversions can be induced at target C without generating bystander mutations at *Tia1*, *Dmd*, and *Tyr-1*, thus precisely mimicking the p.P362L missense mutation of human amyotrophic lateral sclerosis (ALS) [18], the p.Q869Stop nonsense mutation of Duchenne muscular dystrophy (DMD) [19], and the p.Q48Stop nonsense mutation of oculocutaneous albinism type 1 (OCA1) [20], respectively (Fig. 3d). Overall, these results demonstrated that engineered eA3G-BE system can efficiently and precisely induce C-to- T editing in rabbit embryos with sequence preference for CC, suggesting its potential to develop animal models for precisely mimicking human genetic diseases.

### Precise base conversion at *Tyr* to recapitulate human albinism using eA3G-BE

**Fig. 4:**
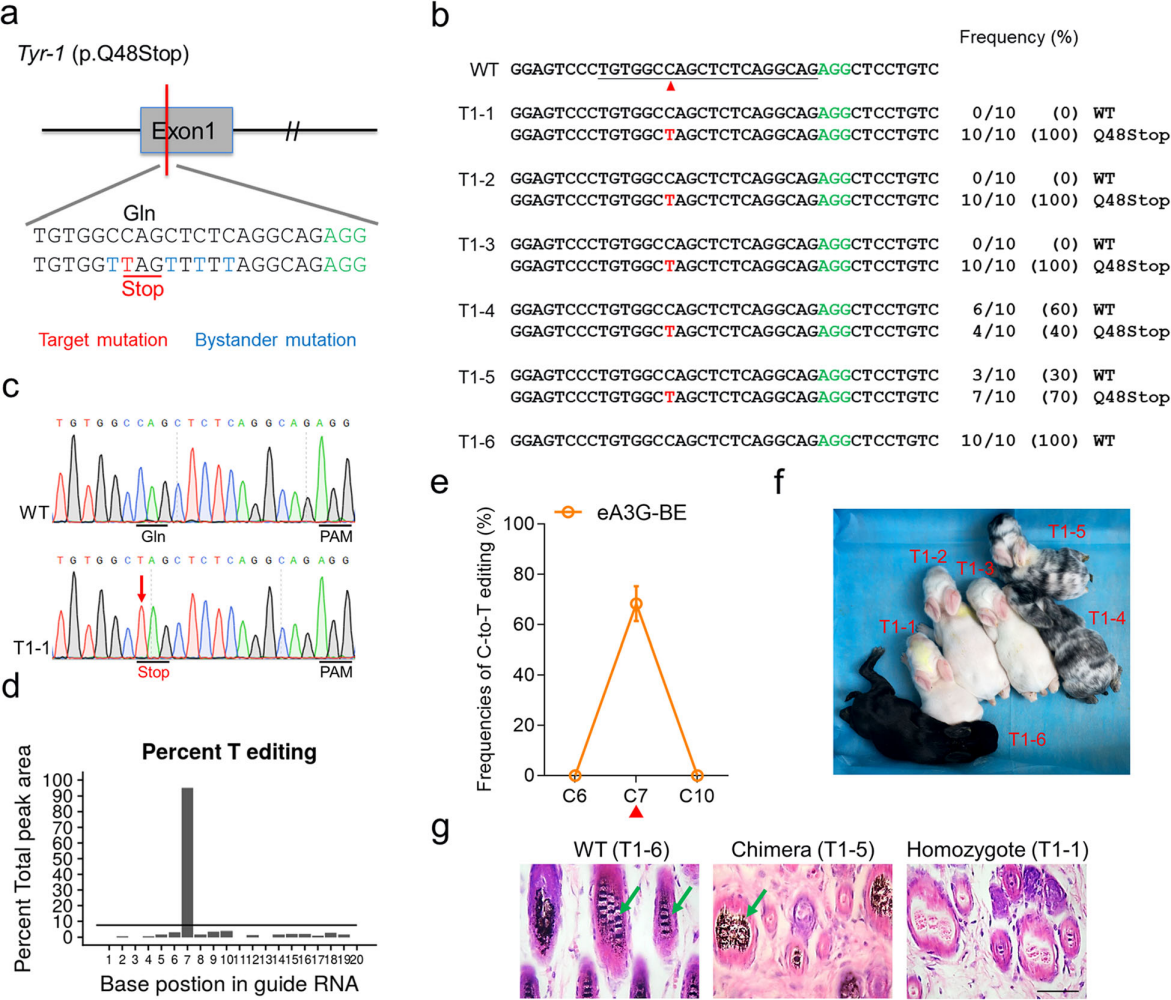
Generation of *Tyr* p.Q48stop rabbits mimic human OCA1 using eA3G-BE system. **a** Target sequence at the *Tyr-1* (p.Q48stop) locus. PAM region (green), target mutation (red), and bystander mutation (blue). **b** Alignments of mutant sequences of F0 rabbits from T-A cloning. The targeted sequence is underlined. The PAM site and base conversions are shown in green and red, respectively. The column on the right indicates frequencies of mutant alleles. WT, wild-type. T1–1 to T1–6, each individual. **c** Representative sequencing chromatograms from a WT and mutant rabbit (T1–1). The red arrow indicates the substituted nucleotide. The relevant codon identities at the target site are presented under the DNA sequence. **d** The predicted editing bar plot based on Sanger sequencing chromatograms from T1–1 by EditR. **e** Summary of single C-to-T editing frequencies of F0 rabbits at *Tyr-1* using eA3G-BE. **f** Photograph of all six F0 rabbits at 1 week. **g** H&E staining of skin from WT (T1–6) and *Tyr-1* mutant (T1–1 and T1–5) rabbits. The green arrows highlight the melanin in the basal layer of the epidermis. Scale bars 50 μm

Subsequently, we further explored the use of eA3G-BE to generate Founder (F0) mutant rabbits. A single C-to-T conversion was designed at *Tyr-1* (p.Q48stop) to mimic human OCA1 in F0 rabbits (Fig. 4a). Rabbit zygotes were transplanted into surrogate mothers after microinjection and six pups were obtained. The result of T-A cloning showed that five of six pups (83%) were mutants with editing frequencies from 40 to 100% (Fig. 4b and Table 1). Notably, targeted base editing at C7 was successfully induced in all of five mutants without any bystander mutations, enabling generation of highly precise p.Q48Stop mutation of OCA1 (Fig. 4b–e). Moreover, three homozygous mutants (T1–1, T1–2, and T1–3) exhibited a complete albino phenotype, and the chimeric mutants (T1–4 and T1–5) exhibited mosaic distribution of black and white skin and hair, which is consistent with their mutant genotype (Fig. 4f). Furthermore, histological H&E staining also revealed the local or complete absence of melanin in the skin of representative T1–5 or T1–1 mutants, but not in the WT rabbit (Fig. 4g). In addition, no apparent off-target mutations were detected at potential off-target sites (POTs) in mutant rabbits, consistent with the results in human cells (Additional file 1: Fig. S5a). Thus, this rabbit model has recapitulated human OCA1 disease symptoms, underscoring the advantageous potentiality of the eA3G-BE system in precisely generating point mutation disease rabbit models.

**Table 1:** Summary of F0 rabbits generated in this study

				Mutant ratio (%)		
System	Target site	Embryos transferred	No. of offspring	No. of mutants	No. of homozygous mutants	No. of bystander mutants
eA3G-BE	*Tyr-1* p.Q48Stop	42	6	5 (83)	3 (50)	0 (0)
eA3G-NG	*Tyr-4* p.W218Stop	38	5	5 (100)	5 (100)	0 (0)

### Expanded targeting scope using eA3G-NG

**Fig. 5:**
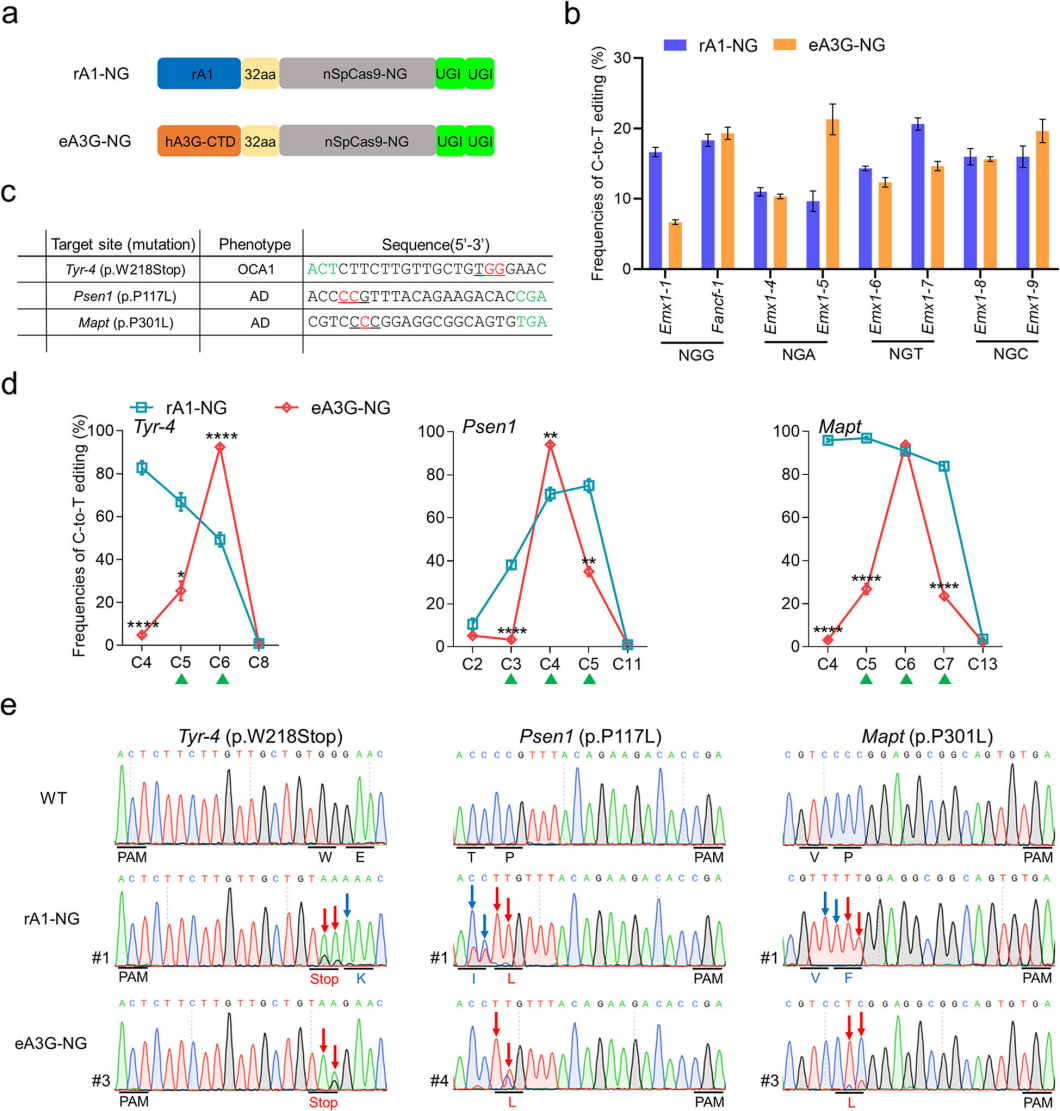
Expanded targeting scope using optimized eA3G-NG fusion in rabbit embryos. **a** Schematic representation of rA1-NG and eA3G-NG architecture. **b** Comparison of the base editing frequencies between rA1-NG and eA3G-NG with NGN PAMs in HEK293T cells. Values and error bars reflect the mean ± s.e.m. of three independent biological replicates. **c** The three target-site sequences within NG PAMs. Target sequence (black), PAM region (green), target base (red), and mutant amino acid (underlined). **d** Comparison of the editing frequencies of single C-to-T conversions between rA1-NG and eA3G-NG at three sites with NG PAMs in rabbit embryos. CCC context (green triangle). *n* = ~ 6 blastocysts. **e** Representative sequencing chromatograms of edited rabbit blastocyst at three target sites using rA1-NG and eA3G-NG systems. Targeted mutations (red arrows) and bystander mutations (blue arrows). The relevant codon identities at the target site are presented under the DNA sequence

The NGG PAM requirement of SpCas9 substantially limits the target sites suitable for eA3G-nCas9 fusions. Therefore, we explore the feasibility of SpCas9-NG system with currently the most relaxed NGN PAMs to expand the genome-targeting scope [21] (Fig. 5a). First, eight target sites with all NGN PAMs were selected to be tested in human cells. Notably, compared with rA1-NG system, eA3G-NG showed comparable efficiency and distinct preference for CC context in all tested sites (Fig. 5b and Additional file 1: Fig. S6). Subsequently, three target sites with NGT or NGA PAM which are arduous to edit using conventional SpCas9-BE were selected to be tested in rabbit embryos (Fig. 5c). Notably, eA3G-NG exhibited high target efficiency comparable to that of rA1-NG at all three sites while significantly reduced bystander activities in non-CCC contexts, which, in turn, substantially decreased unwanted bystander amino acid mutations (Fig. 5d). In particular, with both high precision and expanded space of eA3G-NG, accurate p.P301L mutation can be induced in *Mapt* gene to precisely mimic human classical p.P301L missense mutation of Alzheimer’s disease (AD) [22]. It is extremely difficult for conventional rA1-NG to induce desired p.P301L mutation as it only induces undesired p.P301F mutation due to its high frequencies of bystander C-to-T editing (Fig. 5e).

**Fig. 6:**
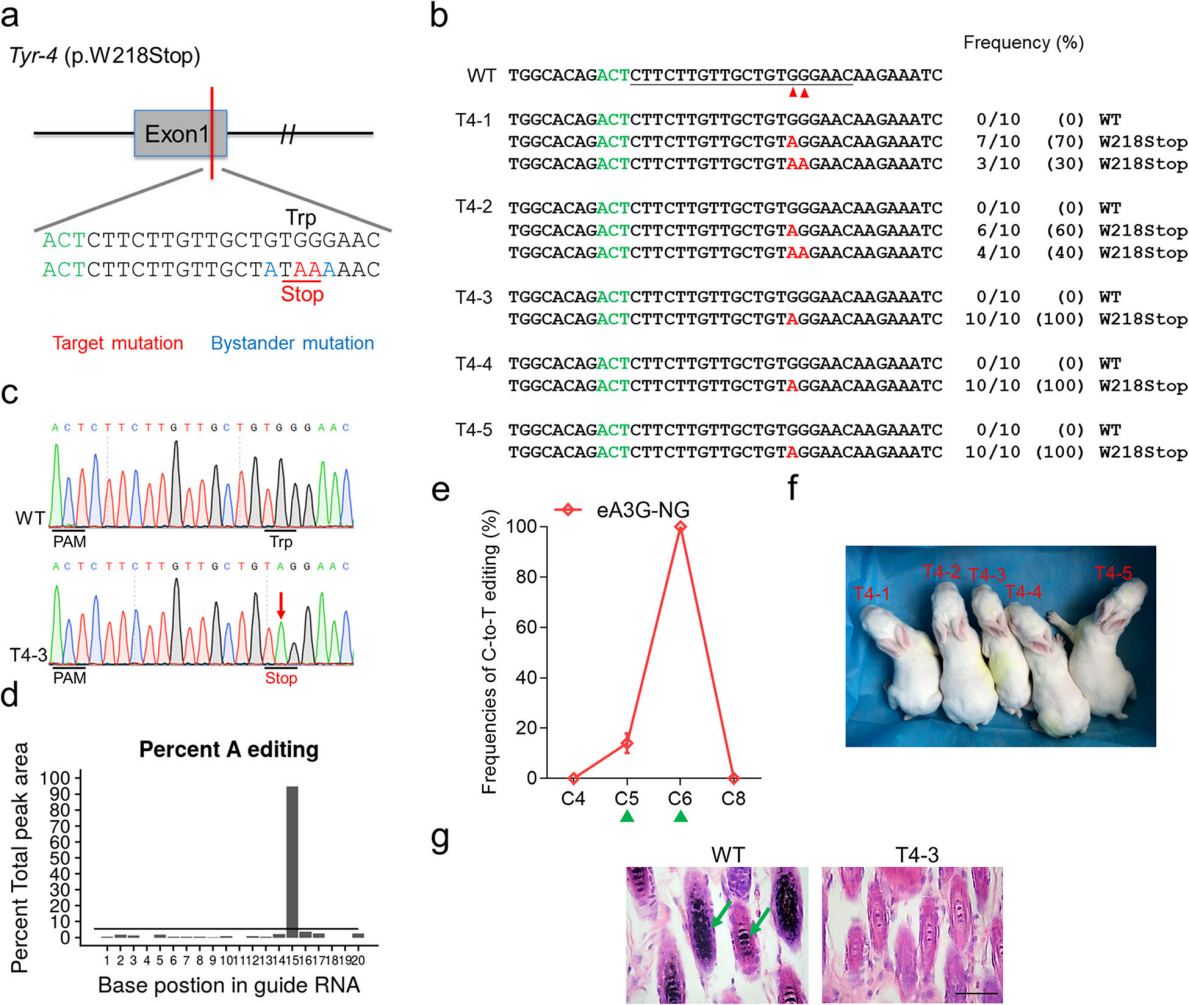
Generation of *Tyr* p.W218Stop rabbits mimic human OCA1 using eA3G-NG system. **a** Target sequence at the *Tyr-4* (p.W218Stop) locus. PAM region (green), target mutation (red), and bystander mutation (blue). **b** Alignments of mutant sequences of F0 rabbits from T-A cloning. The targeted sequence is underlined. The PAM site and base conversions are shown in green and red, respectively. The column on the right indicates frequencies of mutant alleles. WT, wild-type. T4–1 to T4–5, each individual. **c** Representative sequencing chromatograms from a WT and mutant rabbit (T4–3). The red arrow indicates the substituted nucleotide. The relevant codon identities at the target site are presented under the DNA sequence. **d** The predicted editing bar plot based on Sanger sequencing chromatograms from T4–3 by EditR. **e** Summary of single C-to-T editing frequencies of F0 rabbits at *Tyr-4* using eA3G-NG. **f** Photograph of all five F0 rabbits at 1 week. **g** H&E staining of dorsal skin from WT and T4–3 rabbits. The green arrows highlight the melanin in the basal layer of the epidermis of WT rabbit. Scale bars 50 μm

Furthermore, the optimized eA3G-NG was used to generate F0 rabbits that carry the *Tyr-4* (p.W218Stop) mutation in order to mimic human OCA1 (Fig. 6a). Five pups were obtained and all of them (100%) were homozygous with desired nonsense mutation, consistent with the high efficiency in embryos (Fig. 6b and Table 1). Strikingly, no obvious bystander mutations were observed in all rabbits (Fig. 6b–e). All five pups (100%) showed a systemic albino phenotype (Fig. 6f). Moreover, histological H&E staining revealed the absence of melanin in the skin of representative T4–3, but not in the WT rabbit (Fig. 6g). No obvious off-target mutations were detectable at POTs in mutant rabbits (Additional file 1: Fig. S5b). These results indicated the eA3G-NG is highly efficient at relaxed NG PAMs in rabbit and possesses excellent prospects for precisely mimicking human pathogenic point mutations in animal models.

### Loop3 and Loop7 jointly determine the CC context-specificity of eA3G-BE

**Fig. 7:**
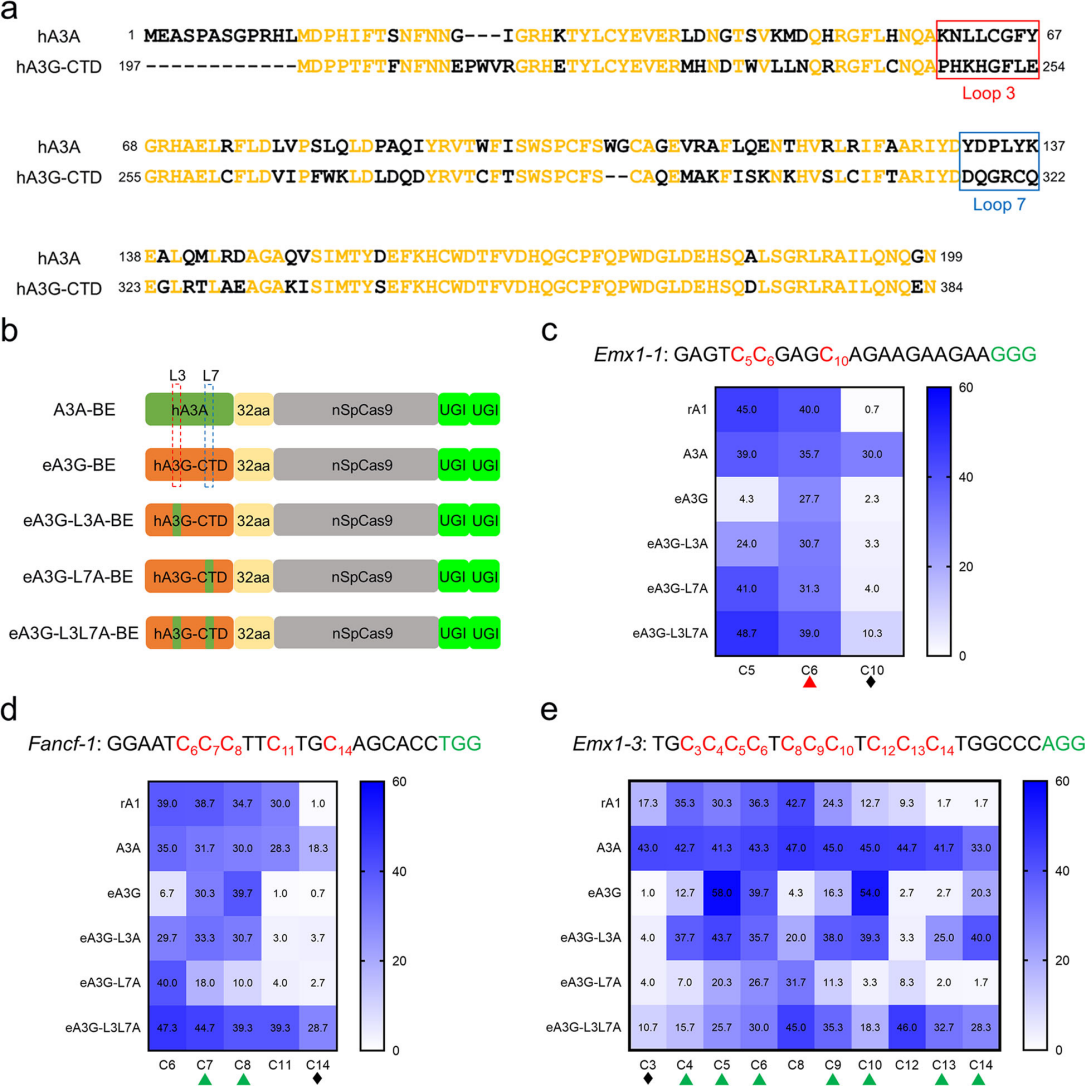
Comparison of base editing activity using loop3 and loop 7 grafted eA3G-BEs. **a** Amino acid alignments of hA3A and hA3G-CTD. The loop 3 and 7 regions are shown in red and blue rectangular box, respectively. **b** Schematic representation of five base editors’ architecture. The dotted rectangle box indicates loop 3 and loop7 region. **c**–**e** Heat maps showing C-to-T editing efficiencies for six base editors at *Emx1-1* (**c**), *Fancf* (**d**), and *Emx1-3* (**e**). The CC or CCC context was shown in red triangle or green triangle, respectively. The GC context was shown in black diamond. Values and error bars reflect the mean ± s.e.m. of three independent biological replicates

It is well known that hA3G is the only cytidine deaminase family member with a unique intrinsic preference for CC dinucleotides in vitro [12, 13]. Our results also confirmed that eA3G-BE maintains a strong CC context-specificity, and we want to explore what determines its unique preference for CC context. In previous reports, hA3A had an intrinsic preference for cytosine preceded by thymine (TC context) in vitro which is different from hA3G [9, 23]. To better understand the differential dinucleotide context-specificity of hA3G and hA3A, we aligned the amino acid sequences of the hA3A and catalytic domain of hA3G (residues 197–384) (Fig. 7a). We found that the protein sequence of hA3A is highly homologous with that of hA3G-CTD, but it is quite different in two key DNA binding loops, loop3 and loop7 (Fig. 7a). In addition, previous study has shown that the loop 3 influences enzymatic activity and loop 7 alone governs the intrinsic preference for CC dinucleotides [24].

Here, to further determine the key loop area responsible for CC context-specificity of eA3G-BE, we constructed a series of chimeras systems, termed eA3G-L3A, eA3G-L7A, and eA3G-L3L7A, in which loop 3 and loop 7 of the hA3G-CTD were replaced with the corresponding loop regions from hA3A (Fig. 7b). Three target sites were tested to directly compare the editing activities of five base editor fusions: the original rA1-BE, A3A-BE, and eA3G-BE and three eA3G-BE variants, eA3G-L3A, eA3G-L7A, and eA3G-L3L7A. Consistent with previous results, the A3A-BE induce similar efficiency compared with that of rA1-BE while showed higher editing activity in a GC context and a larger editing window [10, 15] (Fig. 7c–e and Additional file 1: Fig. S7). It was unexpected that both eA3G-L3A and eA3G-L7A lost original CC context-specificity compared with eA3G-BE in all three tested sites, while with similar or lower overall activity (Fig. 7c–e). In particular, the eA3G-L7A apparently changed the context preference from CC to TC (Fig. 7c–e). Furthermore, the eA3G-L3L7A, which combines both eA3G-L3A and eA3G-L7A, exhibited high editing efficiency, compatibility of GC context, and a large editing window, similar to that of A3A-BE (Fig. 7c–e). Due to its larger editing window and independently of context, the new developed eA3G-L3L7A could initiate base conversions more extensively and increase genome-targeting scope, including the induction of stop (iSTOP) codons and mutation of multiple sites within the gene regulatory regions [25, 26]. Taken together, these results showed that both loop3 and loop7 jointly govern the CC context-specificity of eA3G-BE and simultaneous replacement of loop 3 and loop 7 resulted in a hyperactive eA3G-L3L7A variant similar to A3A-BE, suggesting the potential of developing new and versatile base editors by grafting key loop region.

## Discussion

The base editing precision represents a primary obstacle for base editors, especially for precise disease modeling and gene therapy. Here, we attempted to engineer a fusion of base editors with hA3G, which successfully improved the precision of eA3G-BE with the distinctive preference for CC context. Targeted editing efficiency of 18.3–58.0% or 54.5–92.2% with excellent CC context-specificity was observed in human cells or rabbit embryos, respectively. In addition, by fusing the eA3G-BE with SpCas9-NG, the eA3G-NG with relaxed NG PAMs notably expanded the genome-targeting scope. Moreover, eA3G-BEs were used to induce site-specific single-base substitutions with 83–100% efficiency and few bystander mutations in F0 rabbits at *Tyr-1* p.Q48stop and *Tyr-4* p.W218Stop loci. These data indicated that the efficient and precise eA3G-BEs can be used as a reliable tool for inducing high-precision base editing in rabbits.

Moreover, in this study, we found that both loop 3 and loop 7 jointly determine the CC context-specificity of eA3G-BE. It is worth noting that the loop 3 replacement only enhanced hA3G catalytic activity but did not alter its intrinsic 5′-CC dinucleotide substrate preference in vitro [24]. This divergence may be due to the difference of in vitro and in vivo experimental environment and the influence of the architecture of cytidine deaminase-Cas9 fusion [9]. Taking advantage of deaminase’s natural preference and appropriate artificial design may be a novel perspective to develop new base editing tools. During our preparation of this manuscript, a similar study of engineered APOBEC3G-nCas9 base editors was reported [27]. The editing efficiency in CC context of A3G-BEs was improved through rational engineering A3G deaminase [27]. Therefore, it may be an effective strategy to enhance CC motif preference and increase the editing efficiency of eA3G-BE by changing the key residues.

Additionally, a series of base editors with high precision were developed by narrowing activity window of deaminases, such as YE-rA1 [7], YFE-rA1 [28], truncated CDA1 [8], truncated A3A [29], and rationally designed A3B [30]. Context-dependent base editors, including eA3A-BEs [9] and eA3G-BEs, represent an important advance that offers more precise base editing, while they may lower the target site applicability because the target nucleotide must naturally exist in the preferred sequence context [6]. Therefore, selecting reasonable base editors based on their specific features, such as narrowed window and context-specificity, to preferentially edit the target base over the bystander base so as to obtain desired editing results for the specific sites could be the future trend.

In addition, the requirement of NG PAM still limits the number of target sites suitable for eA3G-BEs. To date, numerous SpCas9 homologs and variants that recognize a variety of PAMs have been found, such as SaCas9 (NNGRRT) [31], Cpf1 (TTTV) [32], NmeCas9 (N4GATT) [33], ScCas9 (NNG) [34], Cas12b (TTN) [35, 36], and Nme2Cas9 (N4CC) [37]. They may further improve the genome-targeting scope of eA3G-BE system when combined with it.

It has been showed that base editors may cause genome-wide off-target DNA and RNA mutations [38–41]. These unexpected off-target DNA and RNA mutations are mainly caused by deaminase domain rather than Cas9 domain. Additionally, the off-target DNA and RNA editing could be eliminated by rational mutagenesis of deaminase domain [42–44]. It has been demonstrated that the Cas9-independent off-targets of hA3G-BE is relatively low due to its CC context-specificity [42]. However, more detailed and diverse examination is required to evaluate eA3G-BEs in future investigation.

The eA3G-BEs can precisely correct point mutations without requiring a DNA-repair template, which makes them as promising tools in gene therapy. However, eA3G-BEs cannot be packaged in a single adeno associated virus (AAV) vector due to AAV packaging limit of ~ 4.7 kbp. It may be solved by using a dual trans-splicing adeno-associated virus (tsAAV) vector system or a split-intein base editor to circumvent the limited cargo capacity of AAV vectors, which have been successfully used to treat many genetic diseases, such as ALS and neurodegenerative ataxia [45–48]. Additionally, finding or designing smaller variants of Cas9 and deaminase is also an effective approach in the future.

## Conclusions

In summary, we develop a series of eA3G-nCas9 fusions that can induce efficient base editing with minimized bystander activity in CC motifs. The eA3G-BE can function as a generic version of the CC context-dependent base editor, and the engineered eA3G-NG further expands genome-targeting scope with relaxed NG PAMs. Thus, these eA3G-nCas9 base editors improve the precision and expand the scope of the currently used rA1-BEs system and have a great potential to be promising tools for precise animal model establishment and gene therapy in the future.

## Methods

### Plasmid construction

The rA1-BE4max was obtained from Addgene (#112093). The DNA fragment of hA3G-CTD, eA3G-L3A, eA3G-L7A, and eA3G-L3L7A was synthesized and cloned into rA1-BE4max by Genscript Biotech (Nanjing). Seven mutations (R1335A/L1111R/D1135V/G1218R/E1219F/A1322R/T1337R) of SpCas9 were introduced into rA1-BE4max and eA3G-BE4max to create rA1-NG and eA3G-NG. Plasmid site-directed mutagenesis was performed using the Fast Site-Directed Mutagenesis Kit (TIANGEN, Beijing). All the site-directed mutation primers are listed in Additional file 1: Table S1. The amino acid sequences of plasmids are listed in Additional file 1: Supplementary sequence.

### Design guidelines of gRNA for eA3G-BEs

The first step in gRNA design is to identify available PAMs (NGG of eA3G-BE and NGN of eA3G-NG) that would place the target C within the major editing window (positions 4–10 in the gRNA target site).

For two-C motifs, eA3G-BEs edit the second C (CC) with similar or lower editing efficiency (site-dependent) compared with rA1-BEs. For three-C motifs, compared with rA1-BEs, eA3G-BEs edit the second C (CCC) with similar or lower editing efficiency (site-dependent) and edit the third C (CCC) with comparable editing efficiency. For motifs containing more than three Cs, eA3G-BEs edit the fourth or more Cs (CCCC) with similar or reduced editing efficiency (site-dependent) compared with rA1-BEs.

### Cell culture and transfection

Human kidney epithelial cell line (HEK293T) were cultured in Dulbecco’s modified Eagle’s medium (DMEM) supplemented with 10% fetal bovine serum (HyClone) and incubated at 37 °C in an atmosphere of 5% CO_2_. The cells were seeded into 6-well plates and transfected using Lipofectamine™ 3000 Reagent (Thermo Fisher Scientific) according to the manufacturer’s instructions. After 72 h, the cells were collected and used for genotyping. All primers used for genotyping are listed in Additional file 1: Table S2.

### mRNA and gRNA preparation

All plasmids were linearized with NotI and transcribed in vitro using the HiScribe™ T7 ARCA mRNA kit (NEB). mRNA was purified using the RNeasy Mini Kit (Qiagen) according to the manufacturer’s protocol. The sgRNA oligos were annealed into pUC57-sgRNA expression vectors containing a T7 promoter. The sgRNAs were then amplified and transcribed in vitro using the MAXIscript T7 kit (Ambion) and purified using the miRNeasy Mini Kit (Qiagen) according to the manufacturer’s protocol.

### Microinjection of rabbit zygotes

The protocol used for the microinjection of pronuclear-stage embryos has been described in detail in our previously published study [49]. Briefly, a mixture of mRNA (200 ng/μl) and sgRNA (50 ng/μl) was co-injected into the cytoplasm of pronuclear-stage zygotes. The injected embryos were transferred into EBSS medium for short-term culture at 38.5 °C, 5% carbon dioxide, and 100% humidity. Then, approximately 30–50 injected zygotes were transferred into the oviducts of recipient rabbit.

### Single-embryo PCR amplification and rabbit genotyping

Each group was injected with an average of approximately 10 embryos to test the base editing efficiency. The injected embryos were transferred into EBSS medium for culture at 38.5 °C, 5% carbon dioxide, and 100% humidity. Then, the injected embryos were collected at the blastocyst stage. Genomic DNA was extracted in embryo lysis buffer (1% NP40) at 56 °C for 60 min, then at 95 °C for 10 min in a BIO-RAD PCR Amplifier. Then, the extracted products were amplified by PCR (95 °C, 5 min for pre-degeneration, 42 cycles of (95 °C, 30 s, 58 °C, 30 s, 72 °C, 30 s), 72 °C, 5 min for extension) and determined by Sanger sequencing. The Sanger sequencing result of each blastocyst was used to evaluate base editing frequencies by EditR [17]. The genomic DNA of newborn rabbits was extracted from ear clips and analyzed by PCR genotyping, Sanger sequencing, and T-A cloning. All primers used for genotyping are listed in Additional file 1: Table S2.

### Off-target assay

The top five potential off-target sites for each gRNA were predicted to analyze site-specific edits according to Cas-OFFinder [50] (http://www.rgenome.net/cas-offinder/). All primers for the off-target assay are listed in Additional file 2: Table S1.

### Hematoxylin and eosin (H&E) staining

The dorsal skin from WT and mutant rabbits was fixed in 4% paraformaldehyde for 48 h, embedded in paraffin wax, and then sectioned for slides. Slides were stained with H&E and viewed under a Nikon ts100 microscope.

### Western blotting

Western blotting analyses were performed, as described previously [51]. The samples from the transfected HEK293T cells were lysed in RIPA buffer supplemented with a protease inhibitor cocktail (Roche, Basel, Switzerland). The antibody against Cas9 (1:1500; ab204448, Abcam) was used as a primary antibody, while tubulin antibody (1:2000; 10094-1-AP, Wuhan Sanying) was used as the loading control.

### Statistical analysis

All data are expressed as mean ± s.e.m. of at least three individual determinations for all experiments. Data were analyzed by Student’s *t* test via GraphPad Prism software 8.0.1. The probability value smaller than 0.05 (*p* < 0.05) is considered to be statistically significant. *p < 0.05, **p < 0.01, ***p < 0.001, ****p < 0.0001.

## Supplementary information

**Additional file 1: Figure S1.** Immunoblots of the rA1-BEs and eA3G-BEs. **Figure S2.** Comparison of base editing frequencies in non-CC contexts with rA1-BE and eA3G-BE. **Figure S3.** Comparison of base editing frequencies at on- and off-target sites with rA1-BE and eA3G-BE. **Figure S4.** Comparison of base editing activity and precision using eA3G-BE and other precise BEs. **Figure S5.** Representative sequencing chromatograms of off-target detection in *Tyr-1* and *Tyr-4* mutant rabbits. **Figure S6.** Comparison of base editing frequencies between rA1-NG and eA3G-NG at 8 target sites with all NGN PAMs. **Figure S7.** Comparison of base editing activity using loop3 and loop 7 grafted eA3G-BEs. **Table S1.** Primers used for site-directed mutation in this study. **Table S2.** Primers used for genotyping in this study. Supplementary sequence. Amino acid sequence of eA3G-BE.

**Additional file 2: Table S1.** The primers used for identifying potential off-target sites in this study.

## Data Availability

The authors state that all data necessary for confirming the conclusions presented in this article are represented fully within the article or can be provided by the authors upon request. All data generated or analyzed during this study are included in this published article and its supplementary information files.

## Abbreviations

CBEs: Cytidine base editors
nCas9: Cas9 nickase
eA3G: Engineered human APOBEC3G
F0: Founder
CRISPR: Clustered regularly interspaced short palindromic repeat
DSBs: Double-strand breaks
indels: Insertion/deletion mutations
SpCas9: *Streptococcus pyogenes* Cas9
PAM: Protospacer adjacent motif
eA3A: Engineered human APOBEC3A
CTD: C-terminal catalytic domain
sgRNAs: Single guide RNAs
ALS: Amyotrophic lateral sclerosis
DMD: Duchenne muscular dystrophy
OCA1: Oculocutaneous albinism type 1
POTs: Potential off-target sites
AD: Alzheimer’s disease
AAV: Adeno-associated virus
