# Supplementary material for: Precise base editing with CC context-specificity using engineered human APOBEC3G-nCas9 fusions
Source: BMC Biol. 2020 Aug 31;18:111. doi: 10.1186/s12915-020-00849-6 (PMC7461344; doi:10.1186/s12915-020-00849-6)
Supplement: Supplementary file 1 — Additional file 1: Figure S1. Immunoblots of the rA1-BEs and eA3G-BEs. Figure S2. Comparison of base editing frequencies in non-CC contexts with rA1-BE and eA3G-BE. Figure S3. Comparison of base editing frequencies at on- and off-target sites with rA1-BE and eA3G-BE. Figure S4. Comparison of base editing activity and precision using eA3G-BE and other precise BEs. Figure S5. Representative sequencing chromatograms of off-target detection in Tyr-1 and Tyr-4 mutant rabbits. Figure S6. Comparison of base editing frequencies between rA1-NG and eA3G-NG at 8 target sites with all NGN PAMs. Figure S7. Comparison of base editing activity using loop3 and loop 7 grafted eA3G-BEs. Table S1. Primers used for site-directed mutation in this study. Table S2. Primers used for genotyping in this study. Supplementary sequence. Amino acid sequence of eA3G-BE. [file 12915_2020_849_MOESM1_ESM.docx]

**
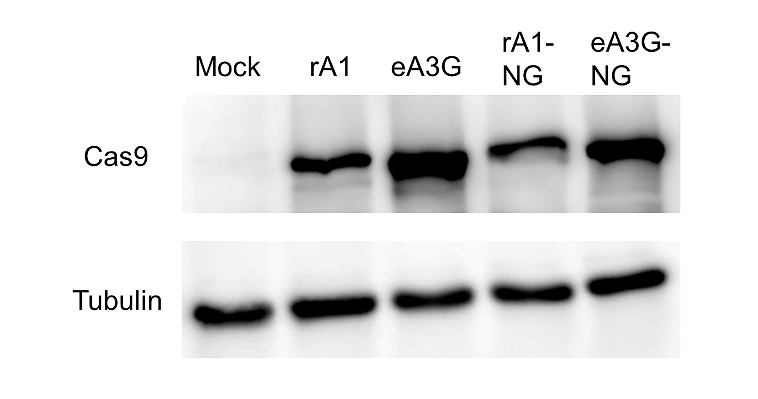
**

**Figure S1**. Immunoblots of the rA1-BEs and eA3G-BEs. Tubulin was used as a loading control.

**
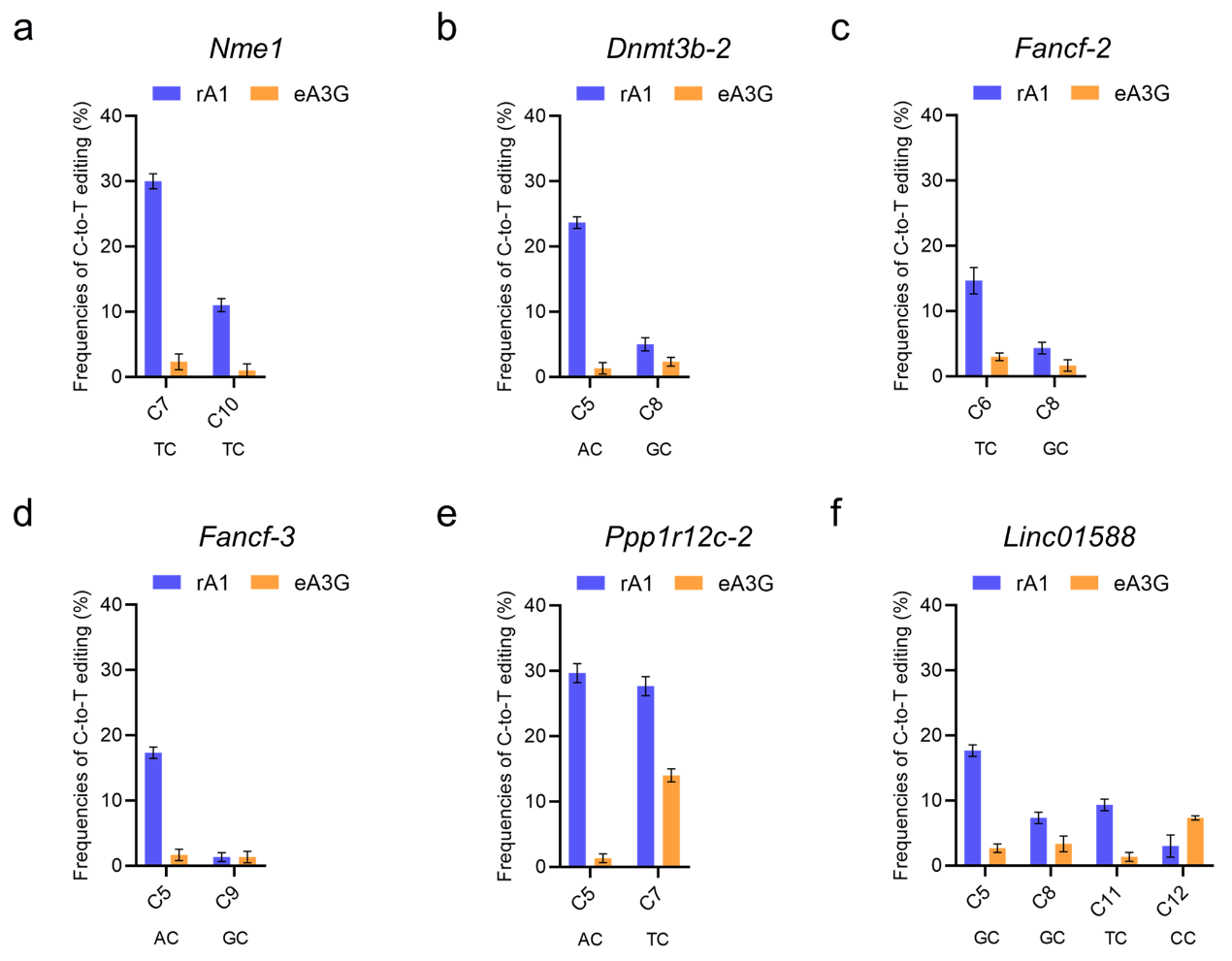
**

**Figure S2**. **a-f** Comparison of base editing frequencies in non-CC contexts with rA1-BE and eA3G-BE at six target sites. Values and error bars reflect the mean ± s.e.m. of three independent biological replicates.

**
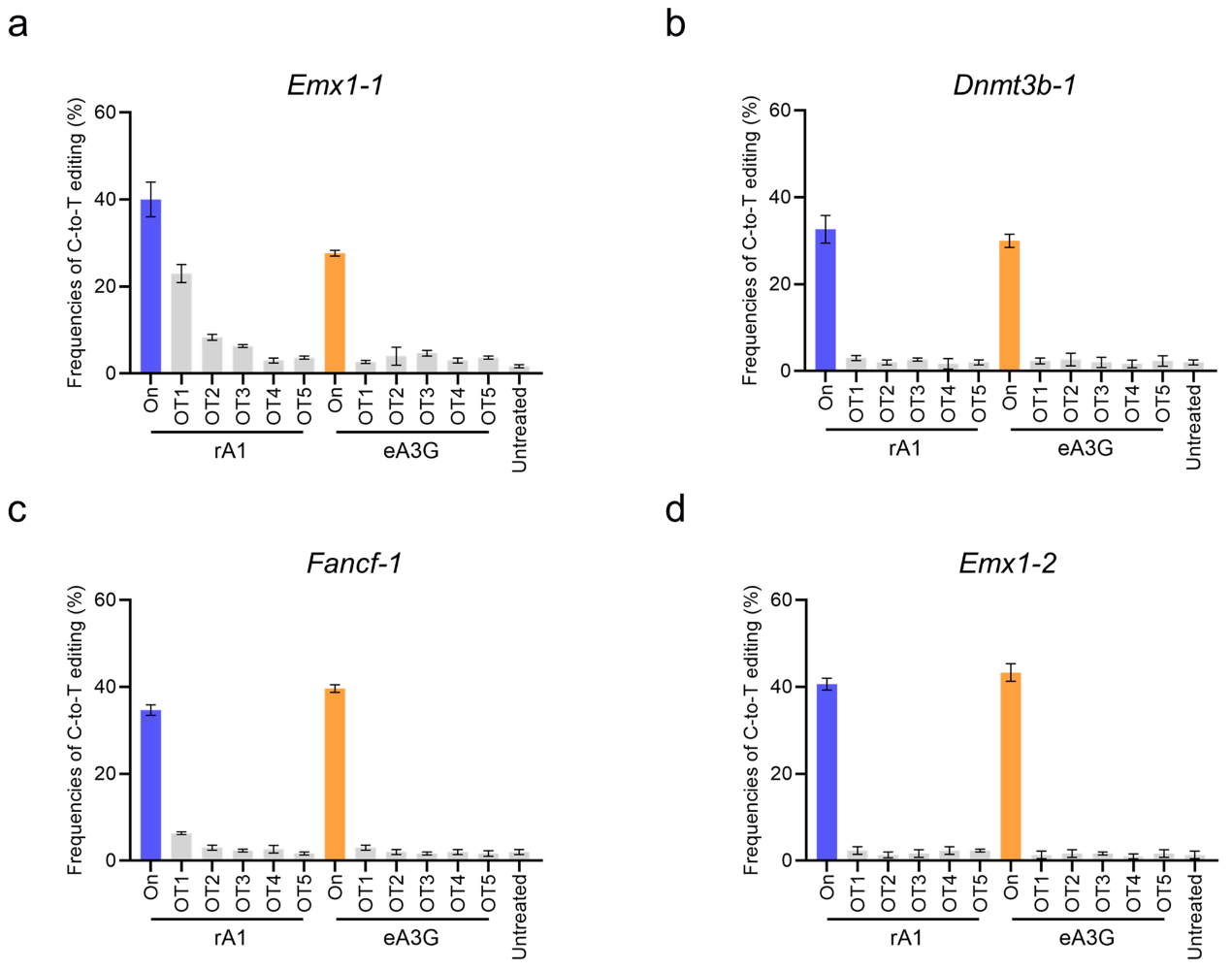
**

**Figure S3**. **a-d** Comparison of base editing frequencies at on- and off-target sites with rA1-BE (left, blue) or eA3G-BE (right, orange) at four target sites. Data for off-target sites are shown in grey. Values and error bars reflect the mean ± s.e.m. of three independent biological replicates.

**
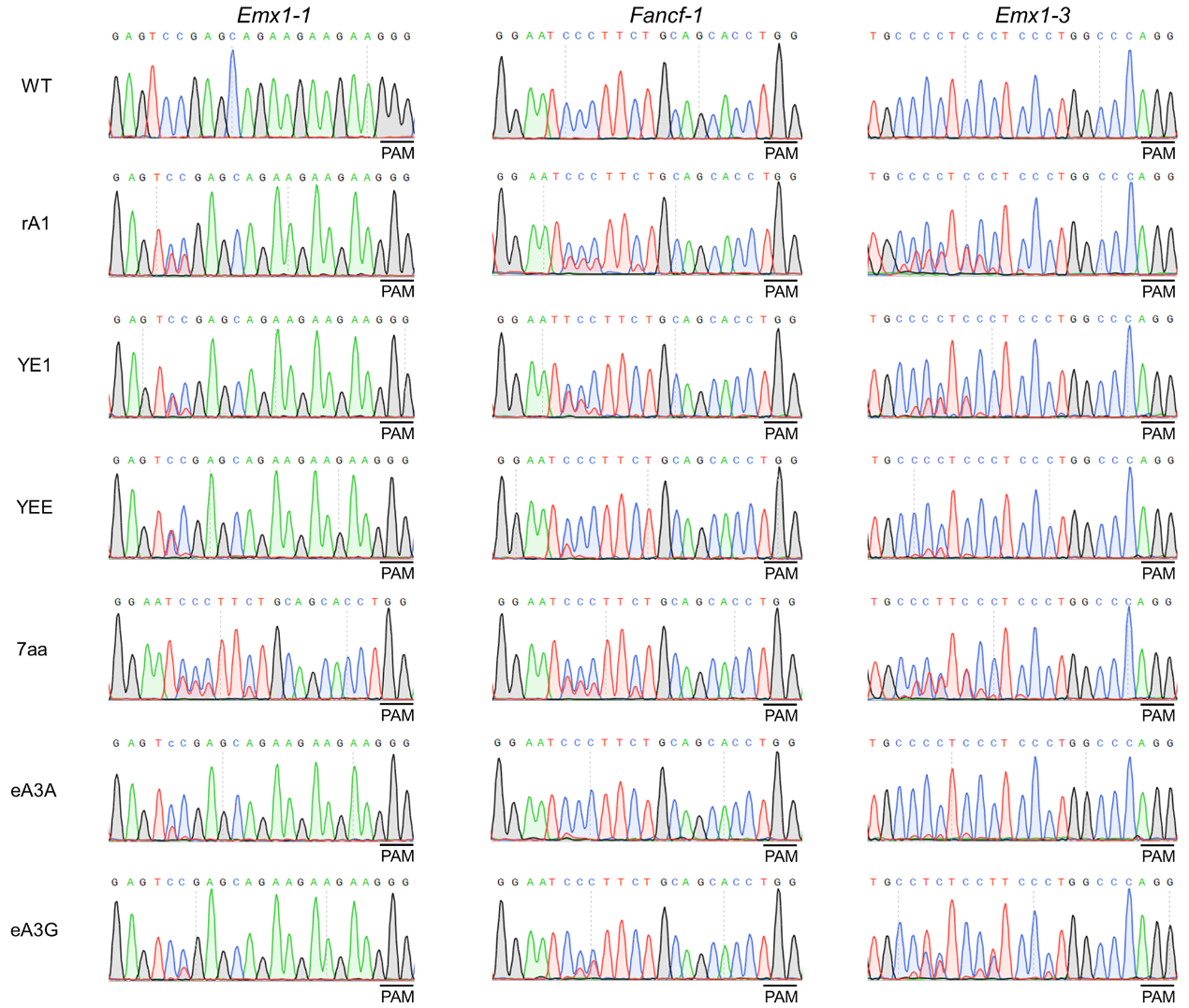
**

**Figure S4.** Comparison of base editing activity and precision using eA3G-BE and other precise BEs. Representative sequencing chromatograms of edited human cells at three target sites using rA1-BE, eA3G-BE and other precise BEs. The PAM (underlined).

**
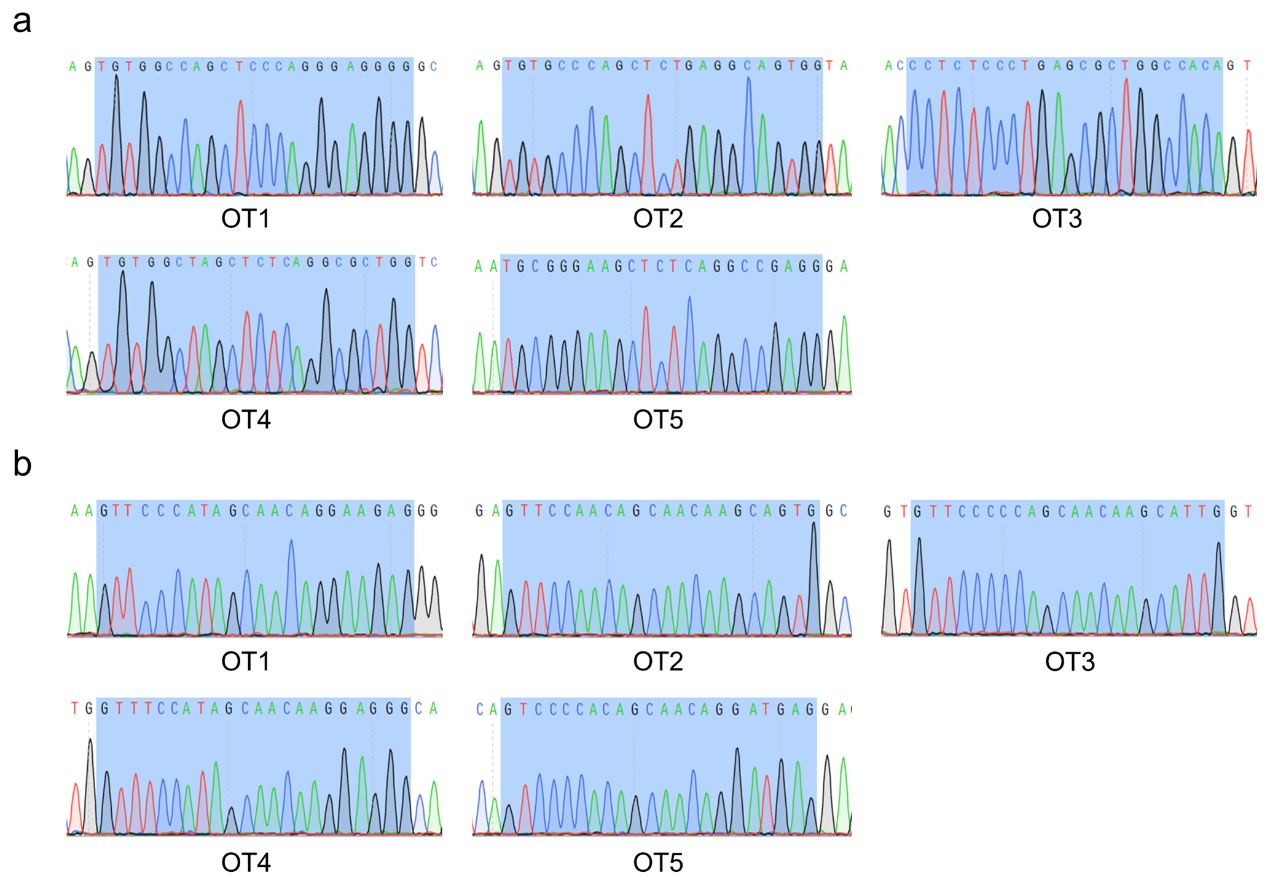
**

**Figure S5.** **a**-**b** Representative sequencing chromatograms of off-target detection in *Tyr-1* (**a**) and *Tyr-4* (**b**) mutant rabbits. Chromatogram sequence analysis of five potential off-target sites (POTs) for sgRNA using PCR products. The 20 bp of the POTs and the PAM are represented in shadow.

**
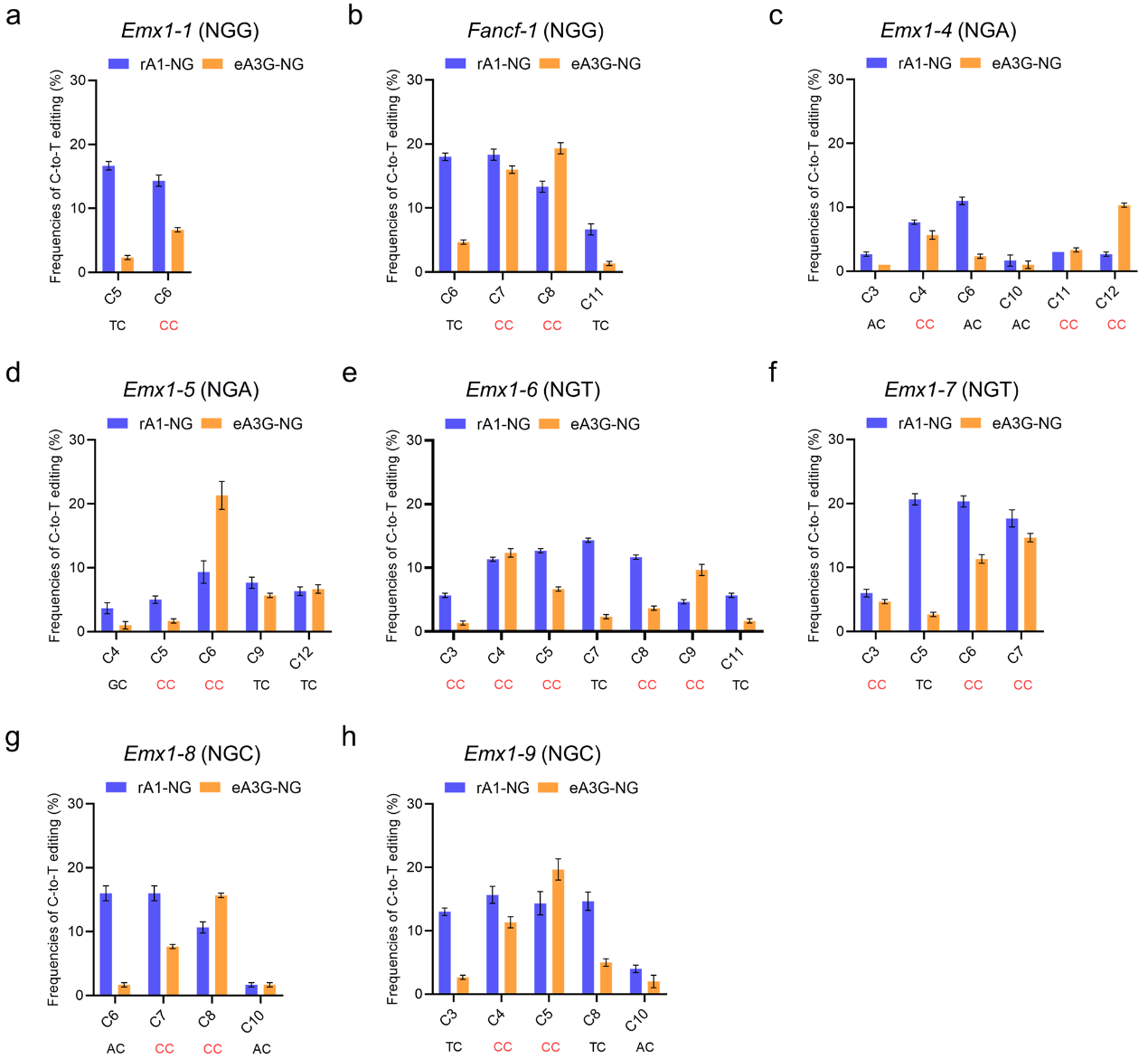
**

**Figure S6**. **a-h** Comparison of base editing frequencies between rA1-NG and eA3G-NG at 8 target sites with all NGN PAMs. Values and error bars reflect the mean ± s.e.m. of three independent biological replicates.

**
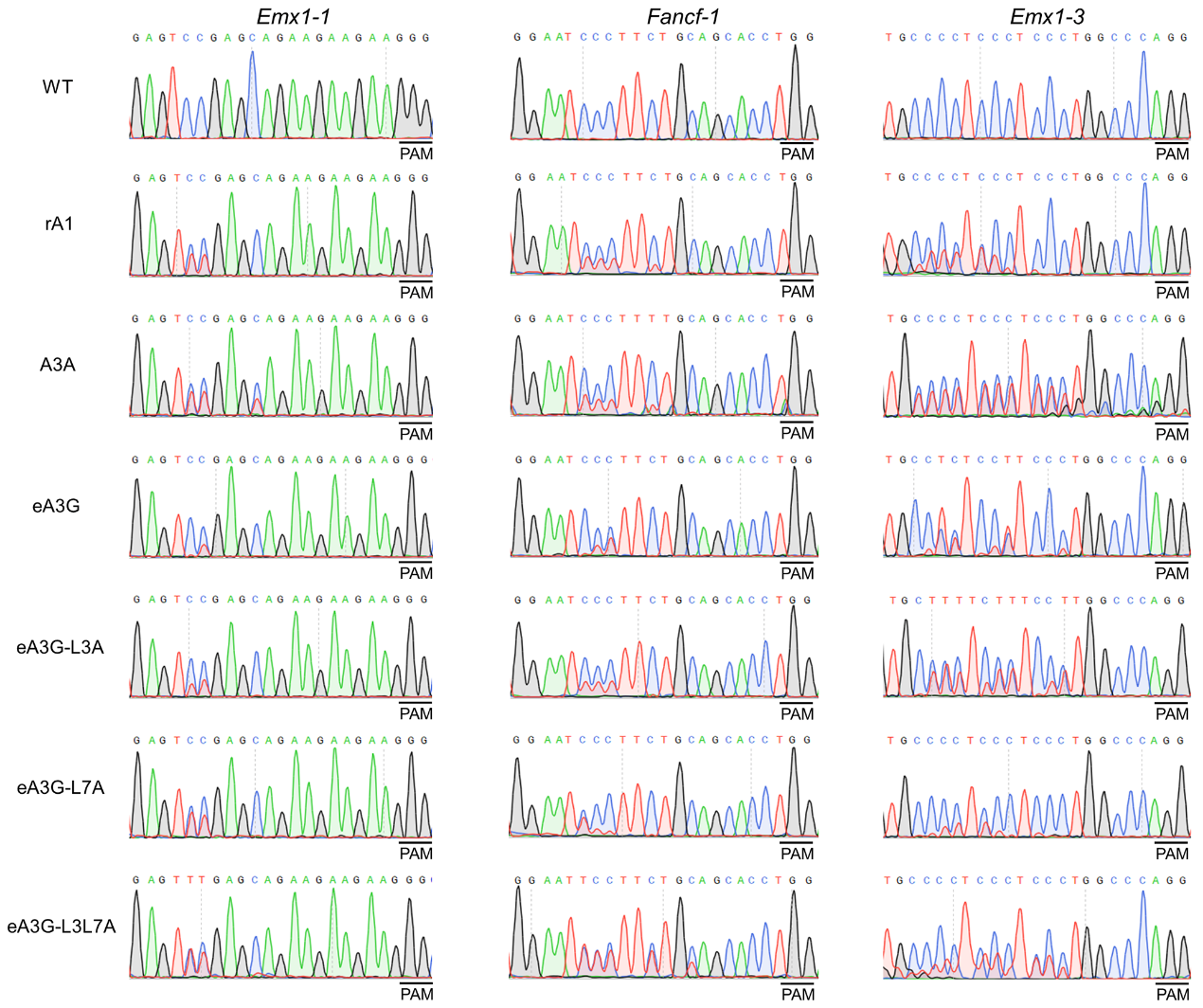
**

**Figure S7.** Comparison of base editing activity using loop3 and loop 7 grafted eA3G-BEs. Representative sequencing chromatograms of edited human cells at three target sites using six BEs. The PAM (underlined).

**Table S1.** Primers used for site-directed mutation in this study.

| **Plasmid template** | **Mutation** | **Primers (5’-3’)** |
| --- | --- | --- |
| rA1-BE and eA3G-BE | L1111R (Cas9) | F: AGCAAAGAGTCTATCCGGCCCAAGAGGAAC  R: CGGATAGACTCTTTGCTGAAGCCGCCTGTC |
|  | D1135V (Cas9) | F: AGTACGGCGGCTTCGTGAGCCCCACCGTGG  R: CACGAAGCCGCCGTACTTCTTAGGGTCCCA |
|  | G1218R,  E1219F (Cas9) | F: GAATGCTGGCCTCTGCCAGATTCCTGCAGAAGGGAAACGA  R: TCGTTTCCCTTCTGCAGGAATCTGGCAGAGGCCAGCATTC |
|  | A1322R (Cas9) | F: AATCTGGGAGCCCCTCGGGCCTTCAAGTAC  R: CCGAGGGGCTCCCAGATTGGTCAGGGTAAA |
|  | R1335A, T1337R (Cas9) | F: CACCATCGACCGGAAGGTGTACCGGAGCACCAAAGAGGTG  R: CACCTCTTTGGTGCTCCGGTACACCTTCCGGTCGATGGTG |

**Table S2.** Primers used for genotyping in this study.

| **Target site** | **Primers** | **Sequence (5’-3’)** | **Product size (bp)** |
| --- | --- | --- | --- |
| *Emx1-1*, *Emx1-3* | F:  R: | CAGCTCAGCCTGAGTGTTGA  CTCGTGGGTTTGTGGTTGC | 277 |
| *Dnmt3b-1* | F:  R: | GGAAGCATTGTTGAGTGGTAGA  CTAGGATACAGCTGCAGAACAC | 488 |
| *Fancf-1* | F:  R: | CATTGCAGAGAGGCGTATCA  GGGGTCCCAGGTGCTGAC | 182 |
| *Emx1-2* | F:  R: | CAGCTCAGCCTGAGTGTTGA  CTCGTGGGTTTGTGGTTGC | 277 |
| *Magea1* | F:  R: | AGAGAAGCGAGGTTTCCATTC  GGTCACAGCAACCTCTGATT | 280 |
| *Ppp1r12c-1* | F:  R: | TACGATGAGTCTCAGAGGACAG  CACACGCACAGGATGAGAA | 245 |
| *Nme1* | F:  R: | GAGGCAGACACACAAACAGA  GTGTGGGAGAACTACAGACATTAG | 281 |
| *Dnmt3b-2* | F:  R: | CCCTTCAAGATGGCTGACAA  TTTCAACCCGAACGGAGAC | 230 |
| *Fancf-2*, *Fancf-3* | F:  R: | AGAGAGTCCTCCTGGAGATTT  ACTACCTACGTCAGCACCT | 428 |
| *Ppp1r12c-2* | F:  R: | AGAGAATGCAGGTCAGAGAAAG  CATGCAGTCCTCCTTACCATC | 345 |
| *Linc01588* | F:  R: | ACCCTGAGAGAGAAAGAGGAA  CCCTGCTTGTGTCAACATAGA | 604 |
| *Emx1-4* - *Emx1-9* | F:  R: | CCTTCTGTGAATGTTAGACCCA  TGCTTGTCCCTCTGTCAATG | 520 |
| *Tia1* | F:  R: | GGCATTACTGTTACGTTGGTATTT GGCAGACATCCAGCATCTT | 459 |
| *Dmd* | F:  R: | TCTTTCAGCCTGTGACTTCAG  GTGGCTTAGCTAAATCTGTAGGA | 421 |
| *Tyr-1*, *Tyr-2* | F:  R: | ATCCGCTCAAGCAGGTATTG  GACATAGTCTGGGCTCGTAGTA | 487 |
| *Fgf5* | F:  R: | CTTCTTCAGCCACCTGATCTT  GGCGCAAGCAACTTACTTAAC | 342 |
| *Tyr-3* | F:  R: | GTGGTGGATGCAAGACTAGAA  AGCTGAAATTGGCAGCTTTG | 408 |
| *Tyr-4* | F:  R: | GCGACTCTTGGTGAGGAAA  AAAGATGCTGGGCTGAGTAG | 459 |
| *Psen1* | F:  R: | TTTACCTAGGGCTCTTGTGTTT  GTGTCTCAGGCTCACCTTATAG | 200 |
| *Mapt* | F:  R: | CCAGACTCTCCCAAGATTCTAATG  ACCCAGGCCGCTTTATTC | 468 |

**Supplementary sequence**

**Amino acid sequence of eA3G-BE.**

Within the sequences below, NLS sequences are in purple, A3G-CTD sequences are in yellow, the SpCas9 nickase sequence is in gray, the UGI sequences are in green.

MKRTADGSEFESPKKKRKVDPPTFTFNFNNEPWVRGRHETYLCYEVERMHNDTWVLLNQRRGFLCNQAPHKHGFLEGRHAELCFLDVIPFWKLDLDQDYRVTCFTSWSPCFSCAQEMAKFISKNKHVSLCIFTARIYDDQGRCQEGLRTLAEAGAKISIMTYSEFKHCWDTFVDHQGCPFQPWDGLDEHSQDLSGRLRAILQNQENSGGSSGGSSGSETPGTSESATPESSGGSSGGSDKKYSIGLAIGTNSVGWAVITDEYKVPSKKFKVLGNTDRHSIKKNLIGALLFDSGETAEATRLKRTARRRYTRRKNRICYLQEIFSNEMAKVDDSFFHRLEESFLVEEDKKHERHPIFGNIVDEVAYHEKYPTIYHLRKKLVDSTDKADLRLIYLALAHMIKFRGHFLIEGDLNPDNSDVDKLFIQLVQTYNQLFEENPINASGVDAKAILSARLSKSRRLENLIAQLPGEKKNGLFGNLIALSLGLTPNFKSNFDLAEDAKLQLSKDTYDDDLDNLLAQIGDQYADLFLAAKNLSDAILLSDILRVNTEITKAPLSASMIKRYDEHHQDLTLLKALVRQQLPEKYKEIFFDQSKNGYAGYIDGGASQEEFYKFIKPILEKMDGTEELLVKLNREDLLRKQRTFDNGSIPHQIHLGELHAILRRQEDFYPFLKDNREKIEKILTFRIPYYVGPLARGNSRFAWMTRKSEETITPWNFEEVVDKGASAQSFIERMTNFDKNLPNEKVLPKHSLLYEYFTVYNELTKVKYVTEGMRKPAFLSGEQKKAIVDLLFKTNRKVTVKQLKEDYFKKIECFDSVEISGVEDRFNASLGTYHDLLKIIKDKDFLDNEENEDILEDIVLTLTLFEDREMIEERLKTYAHLFDDKVMKQLKRRRYTGWGRLSRKLINGIRDKQSGKTILDFLKSDGFANRNFMQLIHDDSLTFKEDIQKAQVSGQGDSLHEHIANLAGSPAIKKGILQTVKVVDELVKVMGRHKPENIVIEMARENQTTQKGQKNSRERMKRIEEGIKELGSQILKEHPVENTQLQNEKLYLYYLQNGRDMYVDQELDINRLSDYDVDHIVPQSFLKDDSIDNKVLTRSDKNRGKSDNVPSEEVVKKMKNYWRQLLNAKLITQRKFDNLTKAERGGLSELDKAGFIKRQLVETRQITKHVAQILDSRMNTKYDENDKLIREVKVITLKSKLVSDFRKDFQFYKVREINNYHHAHDAYLNAVVGTALIKKYPKLESEFVYGDYKVYDVRKMIAKSEQEIGKATAKYFFYSNIMNFFKTEITLANGEIRKRPLIETNGETGEIVWDKGRDFATVRKVLSMPQVNIVKKTEVQTGGFSKESILPKRNSDKLIARKKDWDPKKYGGFDSPTVAYSVLVVAKVEKGKSKKLKSVKELLGITIMERSSFEKNPIDFLEAKGYKEVKKDLIIKLPKYSLFELENGRKRMLASAGELQKGNELALPSKYVNFLYLASHYEKLKGSPEDNEQKQLFVEQHKHYLDEIIEQISEFSKRVILADANLDKVLSAYNKHRDKPIREQAENIIHLFTLTNLGAPAAFKYFDTTIDRKRYTST

KEVLDATLIHQSITGLYETRIDLSQLGGDSGGSGGSGGSTNLSDIIEKETGKQLVIQESILMLPEEVEEVIGNKPESDILVHTAYDESTDENVMLLTSDAPEYKPWALVIQDSNGENKIKMLSGGSGGSGGSTNLSDIIEKETGKQLVIQESILMLPEEVEEVIGNKPESDILVHTAYDESTDENVMLLTSDAPEYKPWALVIQDSNGENKIKMLSGGSKRTADGSEFEPKKKRKV
